# Sub-network transcriptome dataset for diseases associated with exposure to bisphenol F and bisphenol S in human SH-SY5Y neuroblastoma cells

**DOI:** 10.1016/j.dib.2025.111313

**Published:** 2025-01-21

**Authors:** Andrea Guzman, Christina L. Sanchez, Emma Ivantsova, Jacqueline Watkins, Sara Sutton, Christopher L. Souders, Christopher J. Martyniuk

**Affiliations:** Center for Environmental and Human Toxicology, Department of Physiological Sciences, College of Veterinary Medicine, UF Genetics Institute, Interdisciplinary Program in Biomedical Sciences Neuroscience, University of Florida, Gainesville, FL 32611, USA

**Keywords:** Bisphenol replacements, Neurotoxicity, Inflammatory pathways, Protein translation, Protein folding

## Abstract

Bisphenol A replacement chemicals can result in toxicity to neuronal cells, however, the underlying mechanisms are not well characterized. Transcriptome analysis was conducted in the neuronal SH-SY5Y human cell line following exposure of cells to either bisphenol F (BPF) or bisphenol S (BPS) at a concentration of 0.1 nM. Transcriptome data were used to predict which diseases were associated with bisphenol exposure using sub-network enrichment analysis. There were 305 subnetworks perturbed by BPF and 279 subnetworks perturbed by BPS. Top gene sets altered by BPF included urticaria, gastric lesion, attention deficit disorder, familial Mediterranean fever, malocclusion, and lupus erythematosus while for BPS, top gene sets included chronic urticaria, polymyositis, genital herpes, and hypergammaglobulinemia. There were 164 common diseases identified between BPF and BPS datasets. These included protein regulators of androgen deficiency, cerebral toxoplasmosis, metabolic alkalosis, panic attack, T-helper lymphocyte infiltration and vitiligo. Data can be re-used in regulatory toxicology to characterize biomarkers of exposure and elucidate common molecular responses to bisphenol replacements.

Specifications Table

**Table:** 

Subject	Biological Sciences
Specific subject area	Environmental Toxicology and the impact of bisphenol chemicals on neuronal cells
Type of data	Table, figure, and supplemental file of network data in excel
Data collection	Human SH-SY5Y neuroblastoma cells were grown in differentiation media (DMEM:F12 containing 1 % FBS, 1 % antibiotic/antimycotic solution, and 10 µM retinoic acid) for 5 days. On the 6th day, cells were exposed to either media, 0.1 nM BPS, or 0.1 nM BPF for 48 h. RNA was extracted from the cells using TRIzol™ Reagent. RNA-sequencing was conducted on 12 libraries, and the sequencing conducted using a NovoSeq 6000 instrument. The reference genome (homo_sapiens_grch38_p12_gca_000001405_27) and HISAT2 software was used for alignment. Mapping was done using StringTie assembler. Gene expression levels were determined by FPKM and differentially expressed genes were determined. Expression data were inputted into Pathway Studio, and subnetwork enrichment analysis was performed to uncover networks disrupted by either BPF or BPS, relative to untreated cells.
Data source location	Center for Environmental and Human Toxicology, Department of Physiological Sciences, College of Veterinary Medicine, University of Florida, Gainesville, Florida, 32,611, USA
Data accessibility	Repository name: NCBI Gene Expression Omnibus Data identification number: GSE217951 Direct URL to data: https://www.ncbi.nlm.nih.gov/geo/query/acc.cgi?acc=GSM6731081 Direct URL to supplemental data 10.5281/zenodo.14547335
Related research article	None.

## 1 Value of the Data

• Data reveals novel mechanisms underlying toxicity of bisphenol chemicals.
• Transcriptome data provides broader understanding of neuronal cell responses to BPF and BPS.
• Society benefits as toxicity data is used to evaluate new chemicals for safety and exposure limits can be closely monitored in the environment to protect animal and human health.
• Meta-analyses can be conducted using these transcriptome data to uncover common neurotoxicity pathways underlying bisphenol exposure.

## 2 Background

The incorporation of bisphenol A (BPA) in polycarbonate plastics of widely used manufactured products (i.e., plastic wraps, pre-packaged food containers, water bottles) has been gradually phased out after strong evidence supports BPA-induced behavior, immune system, reproductive system, and metabolic dysfunction in wildlife [1,2]. There are also human health concerns for prolonged exposure to BPA as it has been quantified in human urine and tissues [3]. “BPA alternatives” (i.e., bisphenol B, F, G, S, P, Z) are now being utilized extensively in plastic products; however, these analogs share a common chemical structure to BPA and many bind human estrogen receptor (ERs) isoforms with greater affinity than BPA [4]. Additionally, alternatives, such as BPF and BPS, are being detected in similar concentrations in environmental matrices and human urine samples like BPA. Recently, concerns have been raised regarding exposure effects of these alternative chemicals in relation to neurodevelopment and neurotoxicity, as these chemicals have been detected in the human brain [5] and have been found to alter early neural development in children [6,7]. Human SH-SY5Y neuroblastoma cells were exposed to either 0.1 nM BPF or BPS for 48 h. The molecular response in cells was determined using RNA-seq and data collected were subjected to subnetwork enrichment to reveal novel pathways of exposure.

## 3 Data Description

Transcripts were first mapped using [Name + Alias] feature in Pathway Studio, and the sub-network enrichment analysis (SNEA) feature was implemented using differential gene expression data. This approach was conducted to uncover biomarkers of disease associated with either BPF or BPS exposure in human SH-SY5Y neuroblastoma cells. The enrichment p-value for all queries was set at *p* < 0.05.

**Fig. 1:**
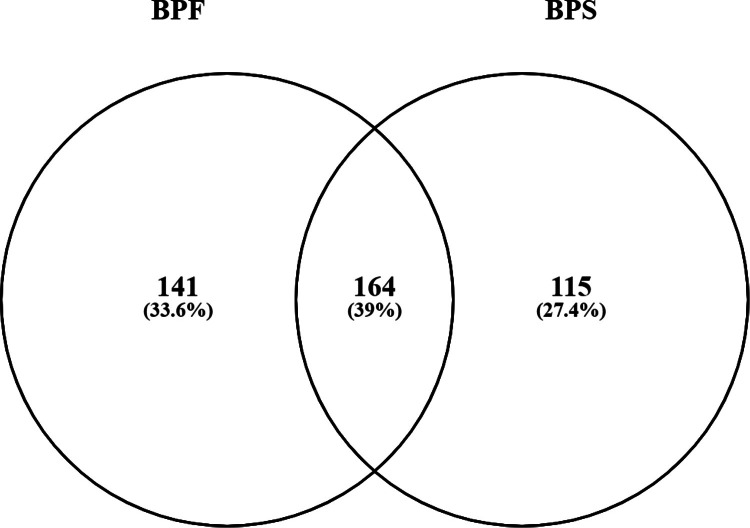
Venn diagram showing the overlap in subnetworks in the BPF and BPS treatment groups.

**Fig. 2:**
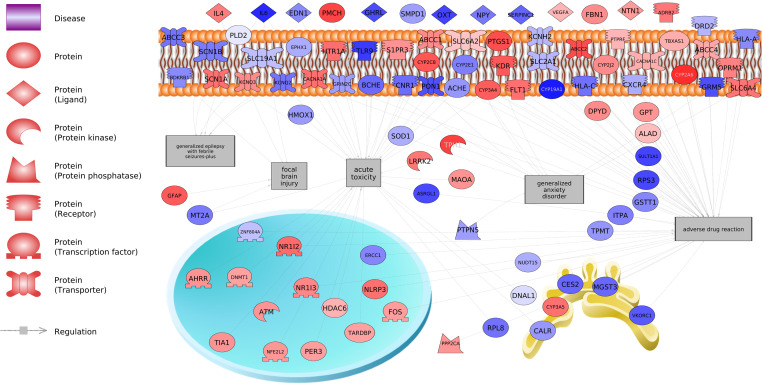
Predicted and combined protein regulators in human SH-SY5Y neuroblastoma cells following exposure to 0.1 nM BPF. The red color entities represent increased expression relative to the control and the blue color entities represent down-regulation of the gene. These data reveal what genes regulate entities enriched in the RNA-seq dataset. Abbreviations of genes and their fold changes are reported in Supplemental Data (doi.org/10.5281/zenodo.14547335).

**Fig. 3:**
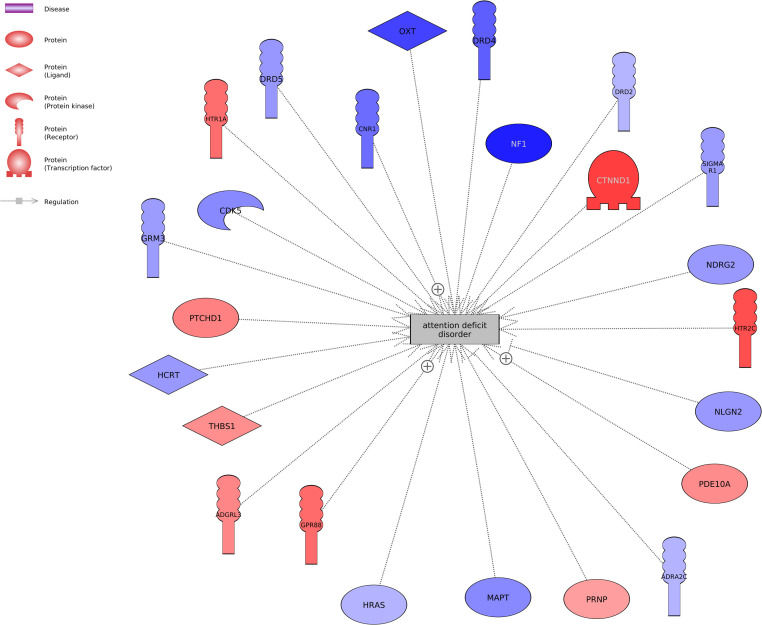
Predicted and combined protein regulators for attention deficit disorder in human SH-SY5Y neuroblastoma cells following exposure to 0.1 nM BPF. The red color entities represent increased expression relative to the control and the blue color entities represent down-regulation. Abbreviations of genes and their fold changes are reported in Supplemental Data (doi.org/10.5281/zenodo.14547335).

Table 1 lists the top disease networks in SH-SY5Y cells treated with either 0.1 nM BPF or BPS (*P* < 0.001). Table 1 presents the gene set seed, total number of neighbors in the network, number of measured neighbors, and median fold change of the network. There were 305 subnetworks perturbed by BPF and 279 subnetworks perturbed by BPS (Supplemental Data, doi.org/10.5281/zenodo.14547335). Of these, there were 164 gene networks for disease that overlapped between the two compounds, which equates to a 39 % overlap in common diseases (Fig. 1). Examples of common protein regulators are urticaria, rhinitis, cardiovascular inflammation, and hyponatremia. Fig. 2 portrays genes related to acute toxicity, adverse drug reaction, focal brain injury, generalized anxiety disorder, and generalized epilepsy with febrile seizures in human SH-SY5Y neuroblastoma cells treated with 0.1 nM BPF. Similarly, Fig. 3 portrays genes associated with attention deficit disorder following human SH-SY5Y neuroblastoma cells treated with 0.1 nM BPF. These pathways connect transcripts based upon the evidence from literature.

**Table 1:** Top disease networks identified in human SH-SY5Y cells exposed to either 0.1 nM BPF or 0.1 nM BPS (*P* < 0.001). The gene set seed, total number of neighbors in the network, number of measured neighbors, and median fold change are provided in the table. Subnetwork output is presented in the Supplemental Data.

Treatment	Gene Set Seed	Total # of Neighbours	# of Measured Neighbours	Median change
0.1 nM BPF	urticaria	80	33	−1.16
	gastric lesion	101	44	−1.05
	familial Mediterranean fever	46	23	−1.38
	malocclusion	20	12	−1.16
	lupus erythematosus	64	30	1.32
	paw edema	115	51	−1.11
	hypergammaglobulinemia	40	19	1.07
	viral myocarditis	142	61	1.09
	genital herpes	28	14	1.17
	chronic urticaria	60	14	1.072

0.1 nM BPS	lupus erythematosus	64	31	1.45
	chronic urticaria	60	16	−1.22
	urticaria	80	31	−1.22
	polymyositis	53	20	1.079
	genital herpes	28	12	1.0004
	hypergammaglobulinemia	40	17	−1.011
	anterior uveitis	45	14	1.58
	gastric lesion	101	41	−1.28
	acquired immunodeficiency syndrome	198	85	1.06
	DRESS syndrome	19	9	−1.70

## 4 Experimental Design, Materials and Methods

We selected 0.1 nM as the concentration to mirror low levels detected in the central nervous system of humans [5]. Forty-eight hours was selected as the exposure duration to allow the cells sufficient time to respond at a transcriptional level to the bisphenol compounds. We have conducted cell toxicity assays using bisphenols and the SH-SY5Y cells did not show signs of cytotoxicity for this dose and duration. Cells were grown in T75 flasks. Fresh differentiation media (DMEM:F12 with 1 % fetal bovine serum, 1 % antibiotic + antimycotic solution, and 10 µM retinoic acid) was provided every 48 h. On the 6th day, cells were exposed to either media, 0.1 nM BPS, or 0.1 nM BPF for 48 h. Media was then removed, and the remaining cells were added into TRIzol™ Reagent and stored at −80 °C for further processing. Total RNA was extracted using TRIzol™ Reagent (Thermo Fisher Scientific, Waltham, MA USA) as per the manufacturer's instruction. A Qubit® 2.0 Fluorometer (ThermoFisher, Grand Island, NY, USA) was used to measure abundance of RNA and the Agilent 2100 Bioanalyzer (Agilent Technologies, Inc.) was used to assess quality of samples. A total of 12 RNA samples were used for RNA-seq library construction (RINs>7).

Libraries and sequencing were conducted by Novogene (Novogene Corporation, Beijing, China). Subnetwork enrichment was performed in Pathway Studio (Elsevier). These details have been previously published by us [8,9]. The reference genome (homo_sapiens_grch38_p12_gca_000) was utilized from the Ensembl genome website browser. All raw and processed transcriptome data are available via the NCBI Gene Expression Omnibus (GEO) database (GSE217951, release date April 2024). These data reveal what proteins regulate entities enriched in the RNA-seq dataset. Abbreviations of proteins and their fold changes are reported in Supplemental Data.

## Limitations

Differentiated SH-SY5Y neuroblastoma were used for these experiments because they produce catecholamines (dopamine and epinephrine) and they are widely used neuronal cells for neurotoxicity in humans [10,11]. We point out that other cell lines used in neurotoxicology may yield additional and differing molecular data regarding the transcriptome response, based on cell phenotype and culturing methods. SNEA is unbiased toward cell type and is based upon literature connections, which may identify molecular pathways that are irrelevent to neurotoxicity (e.g. hematopoietic networks). However, pathways identified by SNEA require experimental validation to confirm biological relevance to neuronal cells. In addition, the transcriptome data presented here represents two different bisphenol compounds, but only a single exposure concentration and time point. Additional concentration and time points would be necessary to further elucidate the temporal and dose-specific responses in neuronal cells, leading to improved understanding of their potential for neurotoxicity.

## Ethics Statement

The authors have read and follow the ethical requirements for publication in Data in Brief and confirming that the current work does not involve human subjects, animal experiments, or any data collected from social media platforms.

## Credit Author Statement

**AG:** Investigation, Writing First Draft. **CLS:** Investigation, Writing First Draft. **EI:** Review and Editing Final Draft. **JW:** Investigation. **SS:** Investigation. **CLSII:** Investigation, Writing First Draft. **CJM:** Investigation and Analysis, Writing, Review and Editing, Supervision, Funding Acquisition.

## Data Availability

NCBI GEOEffects of BPS and BPF on transcriptional networks of neuronal cells (Original data).
